# Thyroid dysfunction: an unlikely culprit behind psychotic symptoms

**DOI:** 10.1192/j.eurpsy.2024.987

**Published:** 2024-08-27

**Authors:** A. S. Pires, M. Pires, I. F. Vaz, S. Mouta, B. Jesus, J. C. Mendes, S. Castro, D. Cruz e Sousa

**Affiliations:** Department of Psychiatry and Mental Health, Local Health Unit, Guarda, Portugal

## Abstract

**Introduction:**

A number of studies have demonstrated that hyperthyroidism increases the prevalence of psychiatric disorders and the likelihood of depressive symptoms, anxiety and hipomania. Apathetic hyperthyroidism is a syndrome, which presents with symptoms of depression, apathy, somnolence or pseudodementia in the absence of the usual symptoms and signs of hyperthyroidism. This condition is more common in the elderly although it has also been described in young adults and adolescents.

In the majority of cases, treatment of hyperthyroidism results in an improvement in neuropsychiatric manifestations in parallel with an improvement of psychical (somatic?) symptoms and psychotropic medication is deemed unnecessary.

Approximately one-third of patients with Graves’ hyperthyroidism are prescribed psychotropic drugs. Sometimes to treat mental symptoms like psychosis or severe agitation, sometimes to treat mental symptoms remaining after amelioration of hyperthyroidism, and sometimes when the diagnosis of Graves’ hyperthyroidism has been missed and the patient is treated as having a primary psychiatric disorder.

**Objectives:**

To present a case of a patient with neuropsychiatric symptoms caused by thyroid dysfunction.

**Methods:**

Case presentation and non-systematic review of existing literature on Pubmed using the following keywords: hyperthyroidism, psychiatric disorders, psychiatric symptoms, depression, psychosis.

**Results:**

We report the case of a 21-year-old female without history of psychiatric illness who presented to the emergency department with somnolence, apathy, cognitive impairment (answering “I don’t know” to most questions), poverty of speech, abulia, perplexity and delusional belief of ruin, in addition to physical symptoms namely alopecia and weight loss. According to her father, she was very active and dynamic person until two days prior, when he started noticing growing apathy, leading to job absenteeism. Urine analysis for elicit drugs was negative.

Investigation for organic disease was undertaken and the blood analysis revealed overt hyperthyroidism.

She was initially treated with aripiprazol. After thyroid dysfunction was identified, she was evaluated by an endocrinologist and started treatment with tiamazol and propanolol, presenting gradual remission of the psychiatric changes. Aripiprazole was discontinued and she was reevaluated in psychiatry consultation after about a month, with complete remission of psychiatric manifestations and normalized thyroid function.

**Conclusions:**

Neuropsychiatric manifestations of thyroid dysfunction are often misdiagnosed as a primary psychiatric disorder. It is necessary to optimize the medical management of these patients in whom the psychiatric symptoms masks a curable organic cause.

**Disclosure of Interest:**

None Declared

